# Diabetes prevalence in patients with takotsubo syndrome in a Polish cohort: the meaning of ‘controls’

**DOI:** 10.1007/s12471-016-0922-7

**Published:** 2016-11-24

**Authors:** J. E. Madias

**Affiliations:** 1 Icahn School of Medicine at Mount Sinai, New York, NY USA; 2 Division of Cardiology, Elmhurst Hospital Center, Elmhurst, NY USA

To the Editor:

I read with great interest the report by Zalewska-Adamiec et al. [1] about the 95 patients (89% female) from the northern-eastern part of Poland who were hospitalised with takotsubo syndrome (TTS) in the years 2008–2012. The authors have contrasted the TTS cohort with 101 female patients with an anterior ST-segment elevation acute myocardial infarction (STEMI), and found that TTS is not a benign condition; it has an in-hospital phase characterised by serious complications, but long-term outcomes at follow-up are better than in patients with STEMI. Similar to the ones with STEMI, the TTS patients had a past medical history of hypertension (63.2% vs. 68.3%), but a lower prevalence of diabetes mellitus (DM) (12.6% vs. 29.7%), and hyperlipidaemia (36.8% vs. 64.4%) [1]. It has recently been reported that the prevalence of DM is low in patients with TTS [2], and it has been postulated that this may have pathophysiological connotations. Accordingly, the underlying DM-mediated peripheral neuropathy, with its attendant autonomic nervous system peripheral neuropathy, exerts a halting/ameliorating influence on the autonomic sympathetic nervous system, whose unbridled surge is thought to trigger TTS. Indeed, even if blood-borne catecholamines are at the roots of TTS, instead of a surge of the autonomic sympathetic nervous system, DM is characterised by attenuated adrenal catecholamine secretion rates [2]. Consequently, it would be contributory if the authors provide us with some details about their 12 patients with DM who suffered from TTS (type of DM, i.e., DM1 vs. DM2, duration of the illness, therapy with insulin and/or oral antidiabetic agents, and presence of DM or autonomic peripheral neuropathy). Also, when one contemplates the pathophysiology of TTS, it would be more appropriate to compare TTS patients with subjects of the general resident population (real ‘controls’), than patients with coronary artery disease, or patients admitted with an acute STEMI. Accordingly, I wonder whether the authors have some information about the prevalence of DM, particularly in women ≥65 years old, in the Polish general population, or the population residing in Bialystok, Podlasie Province.
